# Simultaneous bilateral anterior shoulder dislocation as a result of minimal trauma

**DOI:** 10.11604/pamj.2020.36.94.23757

**Published:** 2020-06-15

**Authors:** Ismail El Antri, Youssef Benyass, Ali Zine

**Affiliations:** 1 Department of Orthopedic Surgery, Military Training Hospital Mohamed V, University Mohamed V, Rabat, Morocco

**Keywords:** Bilateral, anterior, shoulder dislocation

## Abstract

Simultaneous bilateral anterior shoulder dislocation is rare, it often occurs after high energy trauma, we report a case of a 43-years-old male presented with bilateral anterior shoulder dislocation when he tries to stand up from the floor, shoulders in extension, abduction and external rotation. A closed reduction was performed without complications, and one month later the right shoulder was stabilized by open Laterjet technique. The evolution was favorable for both shoulders with no pain, recuperation of the range motion and no recurrence of dislocation. Through this case we underline the value of early diagnosis and appropriate treatment of bilateral anterior shoulder dislocation, to avoid complications and obtain good result.

## Introduction

Unilateral anterior shoulder dislocation is very common in traumatology practice [1]. However simultaneous bilateral anterior shoulder dislocation is quite rare. The few cases described in the literature have occurred after high energy trauma, most often during a sports accident [2]. The following case-report described a simultaneous bilateral anterior shoulder dislocation after a minimal trauma during a domestic accident.

## Patient and observation

**Figure 1:**
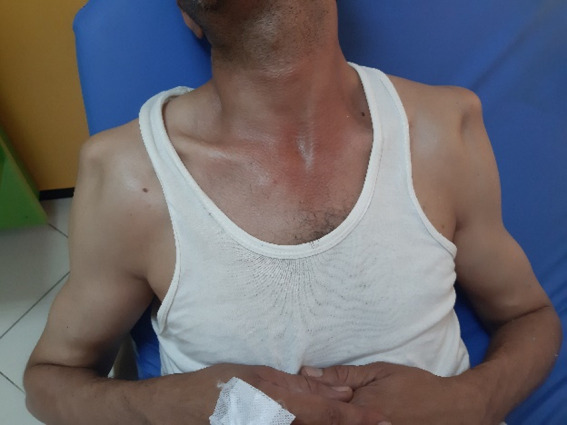
clinical aspect of the anterior bilateral shoulder dislocation

**Figure 2:**
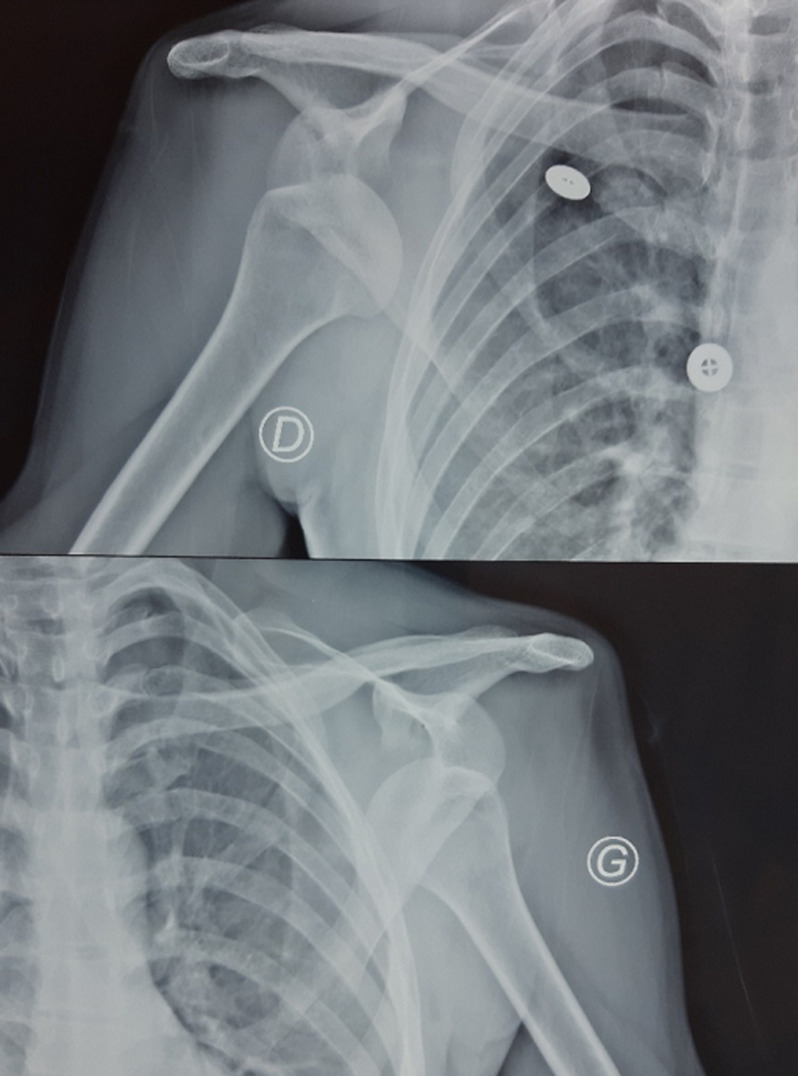
anterior-posterior view of the anterior bilateral shoulder dislocation

**Figure 3:**
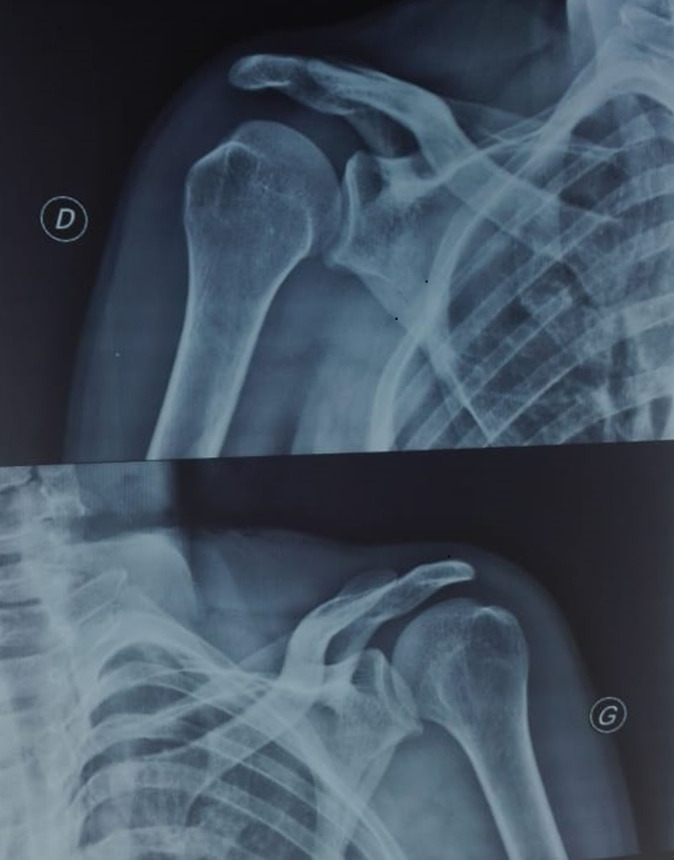
control X-ray after reduction of shoulders dislocation

**Figure 4:**
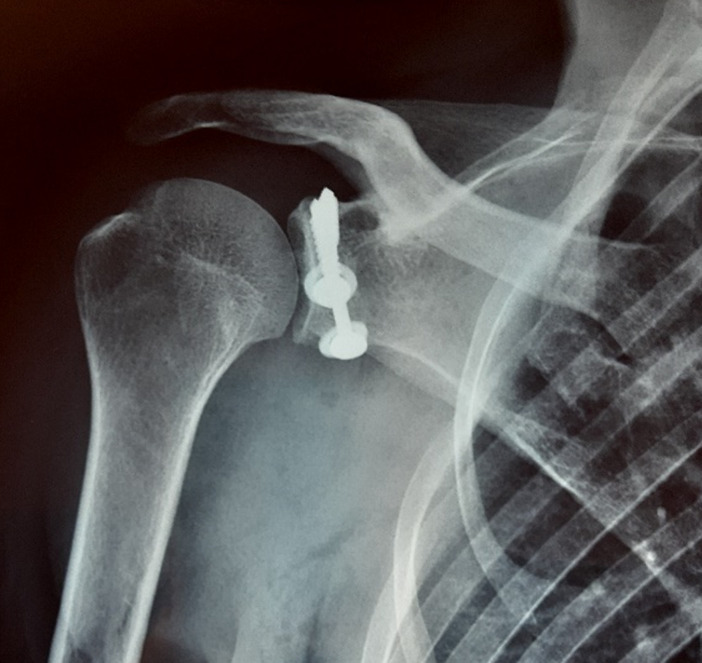
post-operative X-ray of the right shoulder

A 43-year-old male admitted to the emergency department with severe pain in both shoulders and immobility. He was lying on the floor, suddenly he tried to stand up, shoulders in extension, abduction and external rotation. The patient reported two similar accidents in his history, but no surgery, no epilepsy nor hypoglycemia. Physical examination revealed a symmetric deformity with arms held in slight abduction and external rotation, a hollow beneath the acromion and bilateral anterior bulge (Figure 1). Vascular and neurological examinations were normal in both upper limbs. The anteroposterior radiographs in the plane of the scapula confirmed the diagnosis of bilateral anterior shoulder dislocation without associated fractures (Figure 2). A closed reduction by Milch maneuver was performed under sedation, no neurovascular deficit was detected post reduction. Control x-ray showed anatomical reduction without iatrogenic fracture (Figure 3). Both shoulders were immobilized in arm slings for 3 weeks. The patient was referred to the rehabilitation department, after 4 weeks post injury, he recovered a normal range of motion in both shoulders without pain, but he still complained of right shoulder instability with subluxation episodes; therefore, it was decided to stabilize the wright shoulder by open Latarjet technique (Figure 4). Passive mobilization was started immediately during the 2 days of hospitalization, followed by assistive and active mobilization in the rehabilitation department. Active abduction with external rotation of the right shoulder weren´t permitted for 6 weeks after surgery. At follow-up, 6-month post-injury the patient was able to move both shoulder normally, without limitation, pain, or instability.

## Discussion

Bilateral shoulder dislocation is most often posterior, there is a few cases of bilateral anterior shoulder dislocation reported in the literature, they are the result of high energy trauma, most often during high speed sports accident [2, 3]. In our case the trauma was minimal during a domestic accident; therefore, minimal trauma should not rule out this diagnosis. In addition, because of the rarity of this lesion and the fallacious symmetry of the shoulder girdle during clinical examination, the diagnosis of simultaneous bilateral anterior shoulder dislocation may be missed or delayed; Dunlop reported that more than 10% of all bilateral shoulder dislocations are not diagnosed immediately [4], so, clinical examination must be rigorous, followed systematically by x-rays. The latter´s confirm the diagnosis and also allow an assessment, of associated bone lesions, such as proximal humerus fracture, Malgaine notch, and glenoid fracture. After reduction, x-rays are mandatory to confirm the correct position of the humeral heads and rule out iatrogenic fractures. Several techniques of closed reduction of anterior shoulder dislocation have been described in the literature; Alkaduhimi *et al*. gived an overview of 23 different techniques of closed reduction of shoulder dislocation, and 17 modifications of these techniques, they specify that it is not possible to conclude which technique is most successful, provokes less pain, and lead to a minimal number of complications [5]. In our opinion, whatever the technique used, it must be soft, under sedation with total muscle relaxation. The maneuver must be systematically preceded and followed by vascular and neurological examinations of both upper limbs in order to detect possible brachial plexus or axillary artery injury [6]. After reduction the shoulders must be immobilized, in the literature, there is no consensus about duration of immobilization, it varies from 3 to 6 weeks, but in elderly it is admitted that the period of immobilization must be reduced, and physical therapy should be started within 1 week after reduction to avoid shoulder stiffness and muscle wasting. In our case we have used a simple arm sling for 3 weeks, with good clinical outcome, after early rehabilitation. In young patients, like our case, the main complication of anterior shoulder dislocation is the instability of the shoulder; a prospective cohort study reported that 55.7% of young patients developed recurrence of shoulder instability within 2 years [7]. Therefore, it´s necessary to stabilize young patient´s shoulder with surgical treatment, in order to prevent recurrent instability; many options are possible, the most recommended are arthroscopic Bankart and the open Latarjet procedures [8]. The Latarjet procedure involves transplant of the coracoid process to the scapular neck and has demonstrated excellent long-term clinical outcomes and return to sport rate. Recurrent instability is reported to be as low as 0-5.4% [9]. In our case the open Latarjet technique was used with good clinical outcome.

## Conclusion

Simultaneous bilateral anterior shoulder dislocation is a rare situation, it generally occurs after high energy trauma, but can occur also after minimal trauma. Because of the rarity of this lesion, and the fallacious symmetry of the shoulder girdle, clinical examination must be rigorous, followed systematically by x-rays, in order to achieve an early diagnostic and appropriate treatment. Regular follow up and treatment of instability are necessary to prevent excessive damage to the shoulders.
